# Circular RNA circ_0014717 Suppresses Hepatocellular Carcinoma Tumorigenesis Through Regulating miR-668-3p/BTG2 Axis

**DOI:** 10.3389/fonc.2020.592884

**Published:** 2021-01-27

**Authors:** Hongxi Ma, Chunchun Huang, Qiuhuan Huang, Guangzhi Li, Jun Li, Bin Huang, Qiuhong Zhong, Cong Cao

**Affiliations:** ^1^ Clinical Laboratory, Wuzhou Gongren Hospital, Wuzhou, China; ^2^ Department of General Practice, The Affiliated Hospital of Youjiang Medical University for Nationalities, Baise, China; ^3^ Department of Ultrasonics, The Affiliated Hospital of Youjiang Medical University for Nationalities, Baise, China

**Keywords:** hepatocellular carcinoma, circ_0014717, miR-668-3p, B-cell translocation gene 2, ceRNA

## Abstract

Recent studies have reported a close association between circRNAs and cancer development. CircRNAs have been recognized to be involved in various biological processes. Up to now, the function of circRNAs in hepatocellular carcinoma (HCC) is still poorly known. qRT-PCR was used to test circ_0014717 expression in HCC tissue samples and cells was determined. It was shown that circ_0014717 was significantly decreased in HCC. Then, we observed overexpression of circ_0014717 obviously repressed HCC cell growth, migration and invasion. Next, we predicted circ_0014717 acted as a sponge of miR-668-3p. miR-668-3p has been reported to participate in several diseases. In our work, it was shown miR-668-3p was greatly increased in HCC and the direct binding sites between circ_0014717 and miR-668-3p were validated. In addition, B-cell translocation gene 2 (BTG2) is closely involved in cellular carcinogenic processes. BTG2 was predicted as a target for miR-668-3p. By performing rescue assays, we demonstrated that circ_0014717 repressed HCC progression *via* inhibiting BTG2 expression and sponging miR-668-3p. It was manifested loss of circ_0014717 induced HCC progression, which was reversed by BTG2 in Hep3B cells. In conclusion, our findings illustrated a novel circ_0014717/miR-668-3p/BTG2 regulatory signaling pathway in HCC.

## Introduction

Recently, hepatocellular carcinoma (HCC) is becoming a prevalent cancer across the world (1, 2). Meanwhile, in China, HCC contribute a lot to a number of cancer-related death every year (3). In past few decades, great efforts and advances are made in order to improve the treatment for HCC. Despite those, the 5-year survival rate of HCC is still low (4). Herein, it is urgent to identify effective biomarkers for HCC and develop therapeutic targets.

In recent years, circRNAs are attracting increasing attention as a new significant member of non-coding RNAs (5, 6). CircRNAs are covalently closed transcripts derived from precursor mRNA back splicing (7). circRNAs may exhibit specific biological functions and they are significant mediators in tumors through various mechanisms (8–10). For example, circTP63 can act as a ceRNA to induce lung cancer progression *via* inducing FOXM1 (11). CircRNA_100782 contributes to pancreatic carcinoma development *via* activating the IL6-STAT3 pathway (12). CircRNA_5692 represses the progression of HCC *via* sponging miR-328-5p to induce DAB2IP level (13).

Previously, it has been shown that circ_0014717 is obviously reduced in gastric cancer tissues (14). Additionally, in colorectal cancer, circ_0014717 can exhibit tumor-suppressive roles (15). These implied circ_0014717 can serve as an important tumor inhibitor in various cancers. However, the detail role of circ_0014717 in HCC development is barely known.

Currently, the expression of circ_0014717 in HCC was determined. The underlying mechanism of circ_0014717 was investigated by evaluating its downstream microRNA target miR-668-3p and BTG2 in Hep3B and SMMC-7721 cells. In addition, we overexpressed circ_0014717 in HCC cell lines to elucidate *in vitro* and *in vivo* functions. Thus, we hypothesized down-regulated circ_0014717 expression may act as a promising biomarker for HCC *via* regulating miR-668-3p and BTG2.

## Materials and Methods

### Clinical Specimens

30 pairs of HCC and tumor-adjacent tissues were obtained from patients with hepatectomy at The Affiliated Hospital of Youjiang Medical University for Nationalities. No operative treatments, including radiofrequency ablation, immunotherapy or targeted therapy were given to the patients before the surgery. Samples were maintained in liquid nitrogen for RNA and protein isolation. This research was approved by the Ethics Committee of The Affiliated Hospital of Youjiang Medical University for Nationalities, and all subjects gave the written informed consent.

### Cell Culture

LO2, HepG2, Hep3B, Huh7, SMMC-7721, MHCC97L cells were purchased from ATCC (Manassas, VA, USA). DMEM medium (Gibco BRL, Grand Island, NY, USA) was used to incubate the cells. The medium was added with 10% FBS (Gibco BRL, Grand Island, NY, USA) and antibiotics (100 μg/ml streptomycin and 100 U/ml penicillin, Sigma, St-Louis, MO, USA) in a humidified incubator with 5% CO_2_ at 37°C.

### Cell Transfection

Lentivector-mediated shRNA of circ_0014717 (LV-shcirc_0014717) and non-targeting sh-control were synthesized by GeneChem (Shanghai, China). The full-length of circ_0014717 were sub-cloned into the lentivirus vector (LV-circ_0014717) by GeneChem (Shanghai, China). Lentivirus infection was carried out under 8 ng/ml Polybrene. pcDNA3.1-BTG2 and the empty plasmid pcDNA3.1 were obtained from GeneChem (Shanghai, China). miR-668-3p mimics, inhibitors and negative controls were obtained from RiboBio (Guangzhou, China). Lipofectamine 3000 (Invitrogen, Carlsbad, CA, USA) was employed to do cell transfection.

### CCK8 Assay

To carry out CCK8 assay, the transfected cells were seeded into 96-well plates with 3,000 cells in each well. Ten microliters of CCK 8 solution (KeyGene, BioTECH, China) was added to the cells and they were maintained at 37°C for 2 h. The OD values were tested at 450 nm using a SpectraMax microtiter plate reader.

### EdU Assay

EdU assay was carried out using EdU kit (Roche, Indianapolis, IN, USA). Results were obtained using Zeiss fluorescence photomicroscope (Carl Zeiss, Oberkochen, Germany).

### Clone Formation Assay

To carry out clone formation experiment, the transfected cells were seeded in six-well plates. Then, after cells were cultured for 2 weeks, cells were fixed using 30% formaldehyde for 15 min and stained using 0.1% crystal violet (Beyotime Biotechnology, Shanghai, China).

### Flow Cytometry Assay

To carry out cell cycle analysis, cells were fixed by 70% ethanol. 1 ×10^6^ cells were resuspended using 500 μl PI/RNase Staining Buffer. Then, the cells were incubated for 15min with no light. A FACSCanto II flow cytometer (BD biosciences, San Jose, CA, USA) was utilized to analyze cell cycle. To perform cell apoptosis assay, the PE Annexin V Apoptosis Detection Kit I was used. After cells were washed using pre-cold PBS buffer, 5 μl PE Annexin V and 5 μl FITC solution were added to the cells.

### Transwell Assay

To perform the migration assay, cells were trypsinized and then grown in the upper chamber of each insert (Corning, Cambridge, USA) with non-coated membrane with 1% FBS (600 μl). After 24 h, the upper surface of the membrane was removed by using a cotton tip. Cells on the lower surface were stained for half an hour with 0.1% crystal violet. To carry out the invasion assay, matrigel chambers (BD Biosciences, San Jose, CA, USA) were performed. Briefly, cells were collected, re-suspended in medium without serum, and shifted to the hydrated matrigel chambers. The bottom chambers were incubated in 500 μl culture medium with 10% FBS. Then, we scraped the cells on the upper surface, whereas the invasive cells on the lower surface were fixed and colored using 0.1% crystal violet for half an hour.

### Western Blot

Cell lysates were extracted by RIPA buffer added with a protease inhibitor cocktail. Equal amounts of protein samples were separated on 10% SDS-polyacrylamide gel electrophoresis and then transferred onto PVDF membranes (Bio-Rad, CA, USA). After blocked in 5% non-fat dry milk, the PVDF membranes were incubated with the anti-human BTG2 antibody (dilution: 1:1,000, Cell Signaling Technology, MA, USA) and GAPDH antibody (dilution: 1:2,000, Cell Signaling Technology, MA, USA). The protein bonds were visualized by a chemiluminescent detection system (Millipore, Bedford, MA, USA). A FluroChem E Imager (Protein Simple, Santa Clara, CA, USA) was carried out to visualize the western blots.

### Real−Time PCR Analysis

Total RNA of HCC cells and tissues was isolated using TRIzol. 1 µg DNase-treated RNA was reverse transcribed to cDNA using MMLV reverse transcriptase (Takara, Tokyo, Japan). Then, relative quantitative real-time PCR was conducted using SYBR Premix Ex Taq II (Takara, Tokyo, Japan) on LightCycler 96 (Roche, Penzberg, Germany). Then, the expression of target genes was detected using the formula 2^^-ΔΔCt^. The sequences of primers were demonstrated in **Table 1**.

**Table 1:** Primers for real-time PCR.

Genes	Forward (5’-3’)	Reverse (5’-3’)
GAPDH****	GGAGATTGTTGCCATCAACG	TTGGTGGTGCAGGATGCATT
circ_0014717	CTTGCAACATTGCCCTGGATG	CAATGCCTCCCATTGGGTC
****miR-668-3p BTG2**** U6****	TGTCACTCGGCTCGG CATCATCAGCAGGGTGGCG GCTTCGGCAGCACATATACTAAAAT	TGCGTGTCGTGGAGTC CCCAATGCGGTAGGACAC CAGTGCGTGTCGTGGAGT

### RNA Pull-Down Assay

The biotinylated probe was constructed to bind to the junction area of circ_0014717. The circ_0014717 probe (Tsingke, Wuhan, China) was incubated with streptavidin magnetic beads (Life Technologies: Carlsbad, CA, USA). The bound miRNAs in the pull-down materials were extracted using Trizol reagent and qRT-PCR assay was carried out to detect miRNA expression.

### Luciferase Activity Assays

The sequence of 3’-UTR of BTG2 or circ_0014717 were subcloned into pGL3 luciferase reporter vector (Promega, Madison, WI, USA). The WT/MUT 3’-UTR of BTG2 vector or WT/MUT circ_0014717 vector and control mimics or miR-668-3p mimics were co-transfected. The luciferase activity was normalized using a dual luciferase reporter assay system (Promega, Madison, WI, USA).

### Tumor Xenograft

BALB/c nude mice (female, 4- to 5-week-old, 18–20g) were purchased from SLAC (Shanghai, China) and divided into two groups (n = 6 in each group). Hep3B cells (5 ×10^6^ per injection) that were transfected with LV-circ_0014717 and LV-NC, respectively, were implanted into the right flank of the mice. After six weeks, mice were sacrificed under anesthesia. Tumor tissues were subjected to HE and Ki-67 staining. The animal experiments were approved by the Animal Care and Use Committee of The Affiliated Hospital of Youjiang Medical University for Nationalities.

### Statistical Analysis

Data were analyzed using SPSS software 22.0 (Armonk, NY, USA). The Student’s t-test was carried out to analyze differences between two groups. Two-way ANOVA was carried out when more than two groups were compared. Differences were statistically significant if *P <*0.05.

## Results

### circ_0014717 Was Decreased in HCC

**Figure 1:**
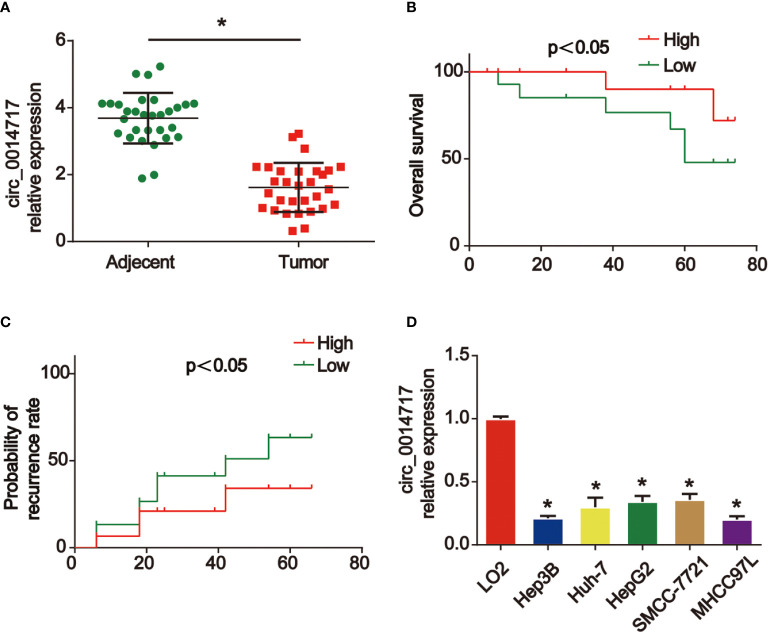
circ_0014717 was increased in hepatocellular carcinoma (HCC). **(A)** The expression of circ_0014717 was tested by real-time PCR in HCC carcinoma and normal adjacent tissues. **(B, C)** Kaplan-Meier analysis of the correlation between circ_0014717 expression and overall survival or recurrence in 30 HCC. **(D)** The expression of circ_0014717 in HCC cells (Huh-7, Hep-3B, HepG2, SMMCC-7721, MHCC97L) and LO2 cells. *P < 0.05.

Firstly, we displayed that circ_0014717 in 30 pair of HCC tissues was greatly lower than that in matched tumor-adjacent tissues confirmed using qRT-PCR analysis in **Figure 1A**. Kaplan–Meier analysis revealed that the expression level of circ_0014717 was significantly associated with the overall survival (OS) and the time to tumor recurrence as displayed in **Figures 1B, C**. In addition, a decreased expression of circ_0014717 was observed in HCC cells (HepG2, Hep3B, SMMC-7721, Huh7 and MHCC97L) in comparison to LO2 cells in **Figure 1D**. We indicated that circ_0014717 down-regulation was a frequent event in HCC. The detailed clinical-pathological parameters of the obtained HCC samples in our study was provided in **Table 2**. Down-regulated circ_0014717 expression level was correlated with tumor size, TNM stage and metastasis.

**Table 2:** Clinicopathological relevance analysis of circ_0014717 in hepatocellular carcinoma (HCC) patients.

circ_0014717				
Feather	Patients	Low expression	High expression	P value
		(<median)	(≥median)	
All cases	30	15	15	
Age, years				0.329
<60	17	7	10	
≥60	13	8	5	
Gender				0.502
Male	20	8	12	
Female	10	4	6	
Tumor size (cm)				**0.002**
≤5	12	5	7	
>5	18	11	7	
TNM stage (I:II:III)				**0.001**
I	13	5	8	
II	8	6	2	
III	9	6	3	
Metastasis				**0.006**
Yes	18	13	5	
No	12	4	8	

For circ_0014717 expression, the median expression level was provided as the cutoff with P-value in bold statistically significant.

### Overexpression of circ_0014717 Suppressed Cell Growth, Migration, and Invasion of HCC Cells

**Figure 2:**
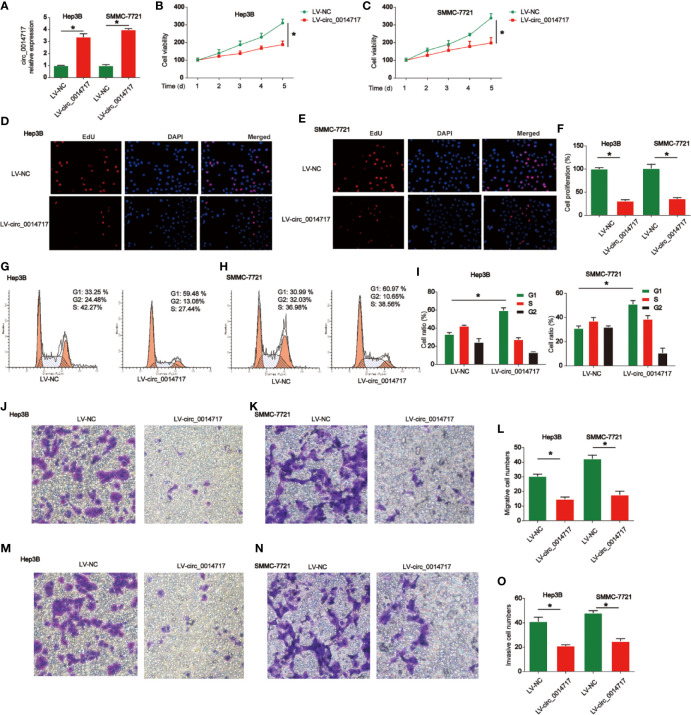
Effects of circ_0014717 overexpression on hepatocellular carcinoma (HCC) cells. **(A)** Expression level of circ_0014717 in HCC cells. Cells were infected with LV-circ_0014717 or LV-NC. **(B, C)** CCK-8 assay was conducted to test cell viability. **(D–F)** Effects of LV-circ_0014717 on HCC cell proliferation evaluated using EdU assay. **(G–I)** Effects of LV-circ_0014717 on HCC cell cycle distribution. Flow cytometry assay was carried out to detect cell cycle. **(J–L)** Effects of LV-circ_0014717 on HCC cell migration. Transwell migration assay was carried out to assess cell migration capacity. **(M–O)** Effects of LV-circ_0014717 on HCC cell invasion. Transwell invasion assay was utilized to detect cell invasion capacity. *P < 0.05.

Next, we studied the roles of circ_0014717 in HCC cell growth. Circ_0014717 was stably increased in Hep3B and SMMC7721 cells as shown in **Figure 2A**. CCK-8 assays indicated overexpression of circ_0014717 repressed the proliferation of Hep3B and SMMC7721 cells (**Figures 2B, C**). EdU incorporation assays also proved that circ_0014717 prominently restrained the growth of Hep3B and SMMC7721 cells (**Figures 2D–F**). Moreover, in **Figures 2G–I**, flow cytometry assays revealed that the cell cycle was blocked by circ_0014717 in G1 phase. Then, in **Figures 2J–L**, transwell migration assay indicated that increased circ_0014717 elevated the migration capacity of HCC cells. In addition, transwell invasion assay was conducted and we found that Hep3B and SMMC7721 cell invasion was significantly repressed by circ_0014717 as demonstrated in **Figures 2M–O**.

### Increased circ_0014717 Depressed Tumorigenesis of HCC *In Vivo*

**Figure 3:**
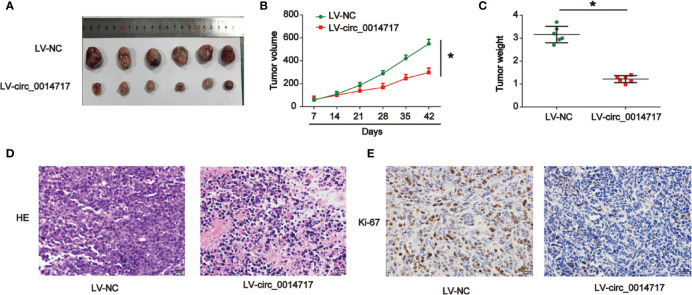
Up-regulation of circ_0014717 restrained hepatocellular carcinoma (HCC)**** cell growth *in vivo*. Twelve 6-week old female BALB/c nude mice were injected with Hep3B cells infected with LV-circ_0014717 or LV-NC. Six mice were used in each group. **(A)** Tumors were peeled from the mice. **(B)** Tumor volume. **(C)** Tumor weight. **(D)** H & E staining. **(E)** IHC staining of Ki-67 in tumor tissues. *P < 0.05.

To investigate the biological roles of circ_0014717 in HCC *in vivo*, Hep3B cells with increased circ_0014717 were implanted into nude mice. The findings of tumor growth curves and tumor weight demonstrated circ_0014717 obviously suppressed tumor growth in mice (**Figures 3A–C**). Subsequently, tumor tissues were harvested for HE and IHC staining in **Figures 3D, E**. Ki-67 assay evidenced that up-regulated circ_0014717 repressed Ki-67 positive cells *in vivo*. Altogether, these suggested that overexpression of circ_0014717 suppressed HCC tumorigenesis *in vivo*.

### Circ_0014717 Acted as a Sponge for miR-668-3p

**Figure 4:**
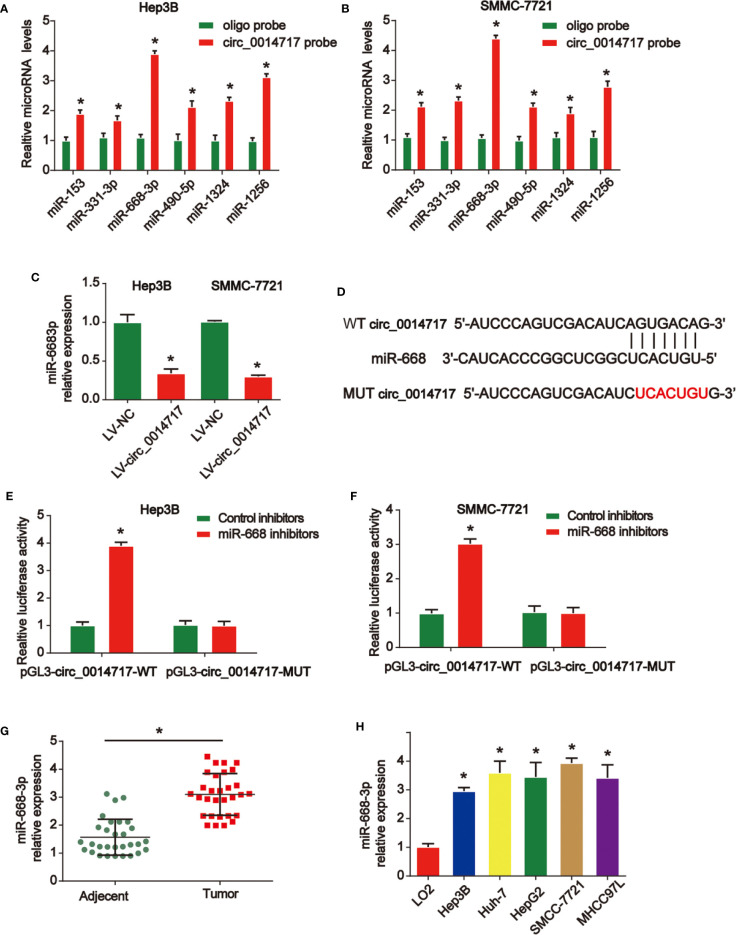
circ_0014717 sponged miR-668-3p in hepatocellular carcinoma (HCC) cells. **(A, B)** Top six miRNA candidates in HCC cell lysates were tested by real-time PCR. Multiple miRNAs were pulled down using circ_0014717 probe. **(C)** The expression level of miR-668-3p in HCC cells infected with LV-circ_0014717 or LV-NC. **(D)** The putative binding sites between circ_0014717 and miR-668-3p. **(E, F)** Luciferase activity was evaluated in HCC cells co-transfected with circ_0014717-WT or circ_0014717-MUT reporter and miR-668-3p inhibitors or its scramble control (NC). **(G)** The expression of miR-668-3p in HCC carcinoma and normal adjacent tissues. **(H)** The expression of miR-668-3p in HCC cells (Huh-7, Hep-3B, HepG2, SMMCC-7721, MHCC97L) and LO2 cells. *P < 0.05.

To explore the mechanisms of the role of circ_0014717 in HCC, as displayed in **Figures 4A, B**, miR-668-3p was most abundantly pulled down by circ_0014717 in HCC cells *via* using https://circinteractome.nia.nih.gov/. Thus, we tested the expression level of miR-668-3p in Hep3B and SMMC7721 cells after circ_0014717 overexpression. Our data revealed that circ_0014717 significantly decreased miR-668-3p expression in HCC cells in **Figure 4C**. Furthermore, the binding sites between circ_0014717 and miR-668-3p was exhibited in **Figure 4D**. Luciferase reporter assay revealed miR-668-3p inhibitors down-regulated the luciferase activity of vectors containing WT-circ_0014717 rather than the vectors containing MUT-circ_0014717 in HCC cells in **Figures 4E, F**. Expression of miR-668-3p in HCC was significantly higher in **Figures 4G, H**.

### BTG2 Was A Novel Target of miR-668-3p

**Figure 5:**
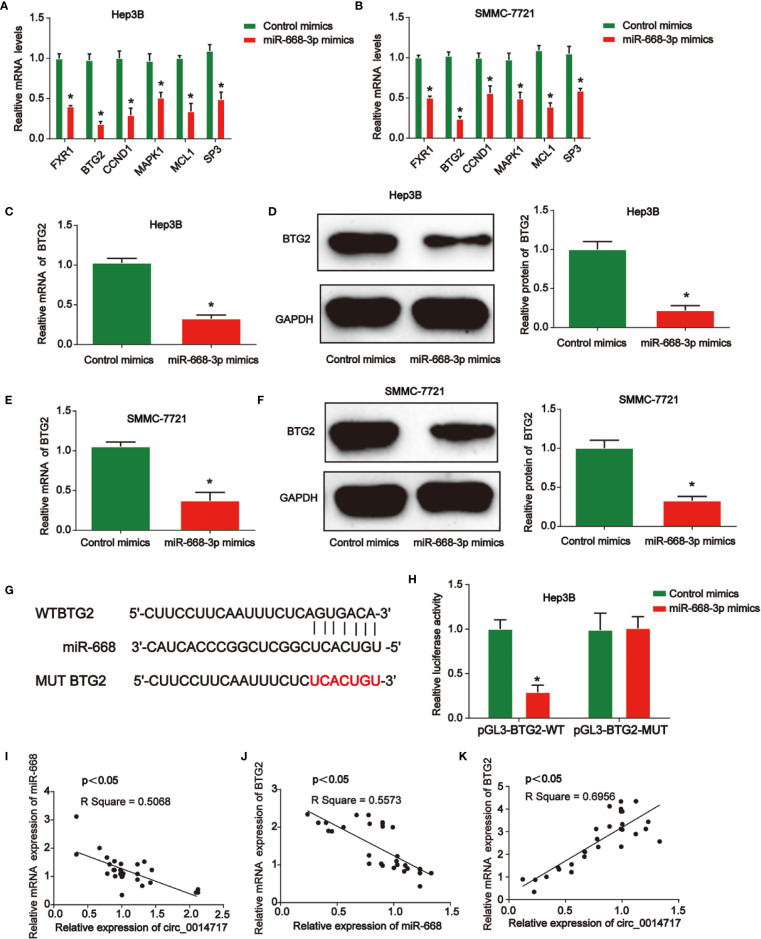
B-cell translocation gene 2 (BTG2) was a direct target of miR-668-3p in hepatocellular carcinoma (HCC) cells. **(A, B)** Top six mRNA candidates in HCC cell lysates regulated by miR-668-3p mimics were tested by real-time PCR. **(C, D)** BTG2 expression in Hep3B cells transfected with miR-668-3p mimics. **(E, F)** BTG2 expression in SMMC-7721 cells transfected with miR-668-3p mimics. **(G)** The putative binding sites between miR-668-3p and BTG2. **(H)** Luciferase activity was evaluated in Hep3B cells co-transfected with BTG2-WT or BTG2-MUT reporter and miR-668-3p mimics or its scramble control (NC). **(I–K)** The correlation of circ_0014717, miR-668-3p, and BTG2 in HCC tissues. *P < 0.05.

Then, we demonstrated the mRNA levels of top six predicted genes after the up-regulation of miR-668-3p in Hep3B and SMMC7721 cells in **Figures 5A, B** *via* consulting online bioinformatics analysis (http://starbase.sysu.edu.cn/). miR-668-3p overexpression depressed the expression of BTG2 in HCC cells most significantly. Then, it was indicated that BTG2 expression in Hep3B and SMMC7721 cells was reduced by miR-668-3p mimics in **Figures 5C–F**. In **Figure 5G**, the putative binding sites between miR-668-3p and BTG2 were exhibited. Then, luciferase activity was evaluated in Hep3B cells co-transfected with BTG2-WT or BTG2-MUT reporter and miR-668-3p mimics. In **Figure 5H**, miR-668-3p mimics induced the luciferase activity of vectors containing WT-BTG2 in Hep3B cells. Next, in **Figures 5I–K**, we verified the correlation of circ_0014717, miR-668-3p, and BTG2 in HCC tissues.

### Restoration of BTG2 Reversed the Effect of circ_0014717 Down-Regulation on HCC Cells

**Figure 6:**
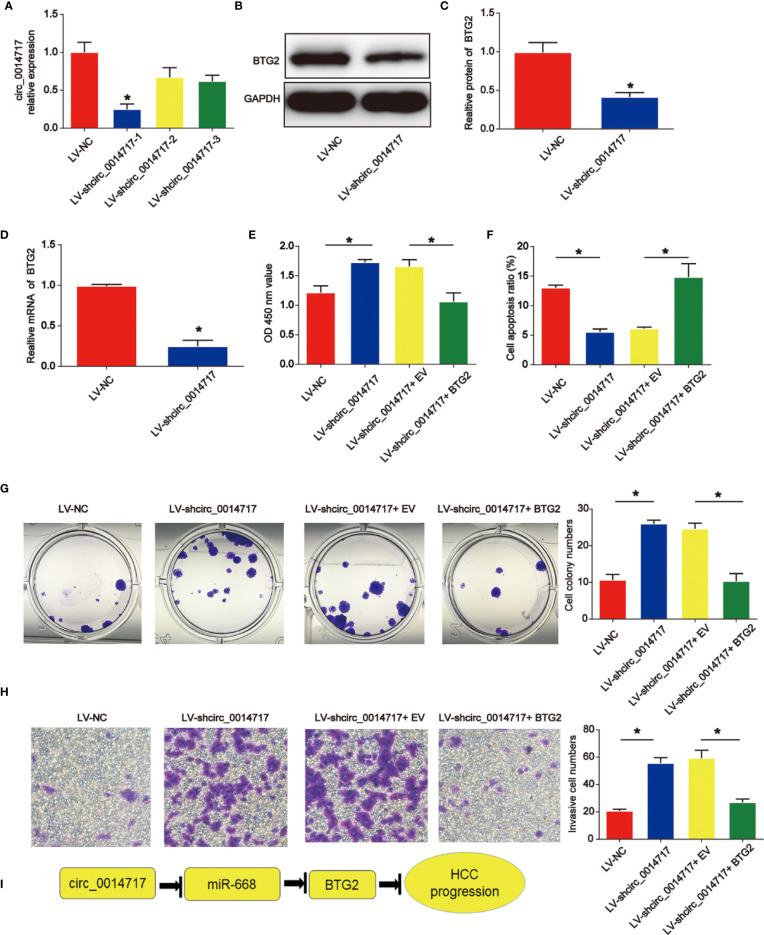
Effects of circ_0014717 shRNA on hepatocellular carcinoma (HCC) cell growth was reversed by B-cell translocation gene 2 (BTG2). **(A)** Circ_0014717 expression in Hep3B cells. Hep3B cells were infected with circ_0014717 shRNA-1, 2 or 3 for 48 h. **(B–D)** BTG2 protein and mRNA expression in Hep3B cells. **(E)** CCK-8 assay was carried out to evaluate Hep3B cell viability. Hep3B cells with decreased circ_0014717 were transfected with BTG2 vector or EV. **(F)** Apoptosis assay was used to assess cell apoptosis. **(G)** Colony formation assay was conducted to test Hep3B cell colony formation capacity. **(H)** Transwell invasion assay was used to detect Hep3B cell invasion. **(I)** Mechanism diagram of circ_0014717/miR-668-3p/BTG2 in HCC progression. *P < 0.05.

To study whether BTG2 was critical for HCC cell proliferation, apoptosis and invasion upon loss of circ_0014717, Hep3B cells with circ_0014717 knockdown were transfected with BTG2 expression vectors. In **Figure 6A**, we confirmed the effect of LV-shcirc_0014717-1 and it exhibited the best knockdown effect in Hep3B cells. LV-shcirc_0014717-1 was used in the following assays. Moreover, we found that BTG2 expression was obviously decreased by circ_0014717 shRNA as exhibited in **Figures 6B–D**. In **Figure 6E**, CCK8 assays proved BTG2 reduced the pro-proliferation role of circ_0014717 knockdown in Hep3B cells. Flow cytometry assays indicated BTG2 overexpression induced apoptosis in Hep3B cells with circ_0014717 knockdown in **Figure 6F**. Furthermore, colony formation also indicated that overexpression of BTG2 reduced the colony formation induced by loss of circ_0014717 as exhibited in **Figure 6G**. Subsequently, Hep3B cell invasion was increased by lack of circ_0014717, which was obviously repressed by the up-regulation of BTG2 in **Figure 6H**. Thus, these results suggested circ_0014717 knockdown triggered HCC cell growth was partially mediated by the repression of BTG2 in HCC. Subsequently, we demonstrated the mechanism diagram of our entire study, which implied that circ_0014717 act as a ceRNA sponge of miR-668-3p to regulate BTG2 expression in HCC progression (**Figure 6I**).

## Discussion

CircRNAs exist in a wide range among various organisms, such as human cells (16). Importantly, since circRNAs are disease-specific, they can function as a potential biomarker for many diseases (17, 18). CircRNAs can manifest many functions, including microRNA sponges, RBP sponges or mRNA masters (19–21).

In our current work, we first reported that circ_0014717 was greatly decreased in HCC tissues and cells. Low circ_0014717 expression levels were associated with significantly reduced overall survival and an increased risk of tumor recurrence. Circ_0014717 might represent an independent prognostic biomarker in HCC patients. Then, overexpression of circ_0014717 was induced in Hep3B and SMMC-7721 cells. We found that circ_0014717 repressed the progression of HCC significantly. Previous studies have confirmed that circ_0014717 is reduced in gastric and colorectal cancer (14, 15). Then, we explored the detailed mechanism of circ_0014717 in HCC progression.

Many studies have investigated the roles of microRNAs in cancers. Many aberrantly expressed miRNAs are reported in the progression of HCC (22–24). As reported, most circRNA molecules have the miRNA response elements, which enable them bind to the corresponding miRNAs. Circ_001569 can induce colorectal cancer growth through targeting miR-145 (25). Circ_RNA circNRIP1 can sponge miRNA-149-5p to promote gastric cancer progression *via* regulating AKT1/mTOR signaling (26). Here, in our work, miR-668-3p was predicted as the target for circ_0014717. miR-668 has been reported to enhance the radio-resistance of breast cancer cell through targeting IκBα expression (27). Here, miR-668-3p was greatly induced in HCC. Result indicated that miR-668-3p and circ_0014717 exhibit opposite expression trends in HCC tissues. Then, we elucidated the direct biological function of the circ_0014717/miR-668-3p axis. We found overexpression of circ_0014717 obviously reduced miR-668-3p expression in HCC cells. This suggested that circ_0014717 may negatively regulate miR-668-3p. Their direct association between them was confirmed in our study for the first time.

Next, BTG2 was found to be the downstream target of miR-668-3p. The function of BTG2 in tumor growth is well explored in several reports (28–30). For instance, BTG2 as a tumor suppressor gene, is upregulated by p53 and PTEN in bladder carcinoma cells (31). In addition, BTG2 is reduced and HCC cancer stem cell-like features are inhibited by BTG2 (32). In our work, we confirm that increased BTG2 reversed the effect of circ_0014717 shRNA on HCC cell growth.

Our investigation has several limitations. The function of the circ_0014717-miR-668-3p-BTG2 axis is warranted to be verified *in vivo*. Although several circ_0014717 and miR-668-3p target genes were predicted using bioinformatics, whether these represent bona fide targets in HCC is still to be studied, which requires more experimental analyses.

In summary, the expression level of circ_0014717 was reduced in HCC. Meanwhile, expression of the downstream target miR-668-3p was negatively correlated with circ_0014717. Additionally, BTG2 was confirmed as the direct target for miR-668-3p. These data uncover a novel circ_0014717-miR-668-3p-BTG2 signaling in HCC cell growth.

## Data Availability Statement

The raw data supporting the conclusions of this article will be made available by the authors, without undue reservation.

## Ethics Statement

The studies involving human participants were reviewed and approved by the Medical Ethics Committee of The Affiliated Hospital of Youjiang Medical University for Nationalities. The patients/participants provided their written informed consent to participate in this study. The animal study was reviewed and approved by the Medical Ethics Committee of The Affiliated Hospital of Youjiang Medical University for Nationalities.

## Author Contributions

HM, CH, and QH performed the experiments and prepared the manuscript. GL and JL collected and analyzed the data. BH and QZ supported administration, technique and materials. CC designed and supervised the study, and revised the manuscript. All authors contributed to the article and approved the submitted version.

## Funding

This study was supported by the National Natural Science Foundation of Guangxi Province (2020JJA140192), the Young and middle-aged teachers’ basic scientific research ability improvement project in Guangxi colleges and universities (No. 2020KY13020), and the Innovation Project of GuangXi Graduate Education (JGY2020166).

## Conflict of Interest

The authors declare that the research was conducted in the absence of any commercial or financial relationships that could be construed as a potential conflict of interest.
